# Exploring Gene Expression Changes in Murine Female Genital Tract Tissues Following Single and Co-Infection with *Nippostrongylus brasiliensis* and Herpes Simplex Virus Type 2

**DOI:** 10.3390/pathogens14080795

**Published:** 2025-08-08

**Authors:** Roxanne Pillay, Pragalathan Naidoo, Zilungile L. Mkhize-Kwitshana

**Affiliations:** 1 Department of Biomedical Sciences, Faculty of Applied and Health Sciences, Mangosuthu University of Technology, Umlazi, Durban 4031, South Africa; 2 Department of Medical Microbiology, School of Laboratory Medicine & Medical Sciences, College of Health Sciences, Nelson R. Mandela School of Medicine Campus, University of KwaZulu-Natal, Durban 4001, South Africa; naidoop5@ukzn.ac.za; 3 Division of Research Capacity Development, South African Medical Research Council (SAMRC), Tygerberg, Cape Town 7505, South Africa; 4 Biomedical Sciences Department of Life and Consumer Sciences, College of Agriculture and Environmental Sciences, University of South Africa, Florida Campus, Johannesburg 1710, South Africa

**Keywords:** mRNA expression, soil-transmitted helminths, herpes simplex virus type 2/HSV-2, *Nippostrongylus brasiliensis*, co-infection, next-generation sequencing

## Abstract

**Background and Aim:** The immunological interactions between soil-transmitted helminths (STHs) and herpes simplex virus type 2 (HSV-2), particularly in the context of co-infection, are poorly understood. Next-generation sequencing (NGS) offers a powerful approach to explore these complex immune responses and uncover potential therapeutic targets. This study leveraged NGS and bioinformatic tools to investigate transcriptional changes and immunological pathways in female genital tract (FGT) tissues of BALB/c mice acutely infected with *Nippostrongylus brasiliensis* (*Nb*), HSV-2, or co-infected. **Methods:** Total RNA was harvested from FGT tissues of BALB/c mice infected with *Nb*, HSV-2, co-infected with both pathogens, and uninfected controls. Differentially expressed genes (DEGs) were identified by comparing uninfected versus infected FGT tissues in R using edgeR and limma packages. Immune-related genes were identified by intersecting DEGs in each group-wise comparison with immune function gene sets derived from the Mouse Genome Informatics (MGI) database. Functional and pathway enrichment analyses were performed with g: Profiler and protein–protein interaction networks were built using the STRING database and visualized with Cytoscape. Key hub genes and significant gene modules were identified using the Cytoscape plugins CytoHubba and MCODE, followed by further functional analysis of these modules. **Results:** NGS analysis revealed distinct gene expression profiles in response to single infection with *Nb* or HSV-2, with both showing significant differences when uninfected controls were compared to infected FGT tissues at a 5% false discovery rate. Notably, there were no significant differences in gene expression profiles between uninfected and co-infected FGT tissues. In the comparison of uninfected versus *Nb*-infected FGT tissues, 368 DEGs were identified, with 356 genes upregulated and 12 downregulated. Several immune-related genes, such as *Ptprc*, *Ccl11*, *Ccr2*, and *Cx3cr1*, were significantly altered. Pathway analysis of DEGs, hub genes, and significant modules indicated modulation of immune and defense responses. Notably, *Nb* infection induced a robust Th2-dominant immune response in the FGT, with downregulation of pro-inflammatory genes. This likely reflects helminth-driven modulation that may impair protective Th1 responses and highlights the systemic impact of *Nb* on the FGT immunity. In the comparison of uninfected versus HSV-2-infected FGT tissues, 140 DEGs were identified, with 121 upregulated and 19 downregulated. Immune-related genes, including *Ldlr*, *Camk1d*, *Lrp8* and *Epg5*, were notably altered. HSV-2 infection led to early and predominant downregulation of immune genes, consistent with viral immune evasion strategies. In addition, functional analysis revealed enrichment in cell cycle and sterol biosynthesis pathways, suggesting that HSV-2 modulates host metabolism to support viral replication while influencing immune responses. In co-infection, no significant transcriptional changes were observed, potentially reflecting immune antagonism where *Nb*-induced Th2 responses may suppress HSV-2-driven Th1 immune responses. **Conclusions:** This preliminary study offers insights into the gene expression responses in the FGT to acute single and co-infection with *Nb* and HSV-2. Together, these findings reveal distinct transcriptomic changes in the FGT following *Nb* and HSV-2 infection, with co-infection potentially leading to immune antagonism and transcriptional equilibrium. This highlights the complex interplay between helminth- and virus-induced immune modulation in shaping FGT immunity. By leveraging NGS, this study highlights important immune-related pathways and serves as a foundation for further investigations into the mechanistic roles of DEGs in immunity to these pathogens, with potential implications for developing novel therapeutic strategies.

## 1. Introduction

Globally, more than 491.5 million people are infected with herpes simplex virus type-2 (HSV-2) [1], while approximately 1.5 billion people are infected with soil-transmitted helminths (STHs) [2]. The highest prevalences of these infections are observed in countries with low socio-economic status and inadequately resourced healthcare facilities, most notably within sub-Saharan Africa [1,3]. Co-infection with HSV-2 and STHs is possible because both pathogens inhabit the same geographical regions, and they can co-exist within the same host [4]. However, immunological effects of co-infection with HSV-2 and STHs are poorly understood as epidemiological and immunological studies are lacking.

Primary infection with genital HSV-2 can manifest as painful genital lesions but is asymptomatic in most individuals [5]. Once infection is established, T-helper type 1 (Th1) HSV-2-specific immunity is induced, engaging both innate and adaptive immune mechanisms. During the early innate immune response, infected genital epithelial cells release type I interferons (IFN-α and IFN-β), which help suppress viral replication and attract immune cells to the site of infection. Natural killer (NK) cells target and eliminate virally infected cells early, and pro-inflammatory cytokines, such as IL-1, IL-6, and TNF-α, promote inflammation and recruit neutrophils and macrophages [6]. Dendritic cells and other innate immune cells detect the virus, activating adaptive immunity by presenting antigens to lymphocytes [7]. Adaptive immunity features IFN-γ-producing CD4^+^ T cells, which boost macrophage and cytotoxic T cell activity, and CD8^+^ T cells, which directly target and eliminate infected cells through viral antigen recognition [6]. Additionally, B cells produce neutralizing antibodies (IgG and IgA) to block viral entry and mediate antibody-dependent cellular cytotoxicity, collectively limiting viral spread. However, HSV-2 is capable of evading immune detection through several mechanisms, including suppressing MHC class I expression to reduce cytotoxic T lymphocyte activity and producing proteins that block type I IFN signaling, thereby dampening antiviral responses [6,7,8]. Additionally, during primary infection, the virus travels to the sensory neurons and ganglia, where it establishes lifelong latent infection. As a result, while the immune response can control viral replication and limit reactivation, it is unable to completely eradicate the virus, leading to recurrent infections [5].

In contrast, STHs primarily induce a dominant T-helper type 2 (Th2) immune response, comprising both innate and adaptive immune mechanisms. The innate response to STHs comprises the release of cytokines, such as IL-4, IL-5, and IL-13, which drive eosinophilia, mast cell activation, and IgE production, which are key mechanisms required for the expulsion of parasites. IL-13 stimulates mucus production and the contraction of intestinal smooth muscles, aiding in parasite expulsion, while IL-5 enhances eosinophil activity to kill larvae or adult worms [9,10]. Additionally, adaptive immunity further supports this process by promoting Th2 cell development and associated cytokine secretion, facilitating parasite clearance and reducing the risk of reinfection [9]. Moreover, regulatory T and B cell populations, and alternately activated macrophages reduce excessive inflammation, creating a hyporesponsive and immune-tolerant environment by producing anti-inflammatory cytokines, such as IL-10, and transforming growth factor beta (TGF-β). Consequently, STHs often modulate host immunity to bystander infections [11].

STHs can modulate host immune responses to sexually transmitted viral infections. For example, in individuals co-infected with STHs and human immunodeficiency virus (HIV), the adverse effects associated with HIV may be intensified due to reduced CD4^+^ cell counts, disrupted immune cell function, and elevated HIV viral loads [12,13]. Similarly, hookworm infection was linked to an increased risk of human papillomavirus (HPV) infection and elevated HPV viral loads, and mixed type 1/type 2 immune responses were observed in the vaginal tracts of STH/HPV co-infected women [14,15,16]. Co-infection with STHs and HSV-2, however, is less understood. Although limited, experimental studies have reported that in co-infected female mice, *Nippostrongylus brasiliensis* (*Nb*), a close relative of the human hookworms, exacerbated genital HSV-2. Co-infection with *Nb* and HSV-2 resulted in heightened vaginal ulceration and was associated with higher levels of IL-5 and IL-13 and the accumulation of eosinophils in the vaginal tract [17]. However, there is a lack of data on the immunological interactions at the mRNA gene expression level in STH/HSV-2 co-infected individuals. To address this gap and recognizing that the molecular mechanisms underpinning these interactions remain poorly understood, this preliminary study sought to investigate and identify the major transcriptomic features associated with *Nb* and HSV-2 single- and co-infection, using next-generation sequencing (NGS). Herein, we describe changes in gene expression profiles of the murine female genital tract following acute *Nb* and HSV-2 single and co-infection. Understanding the immunological interactions between STHs and HSV-2 at a genomic level is relevant to effective infection control and elimination.

## 2. Materials and Methods

### 2.1. Ethical Approval

Ethical clearance for the study was obtained from the Animal Ethics Committee at the University of Cape Town (UCT) (reference number: FHS AEC REF NO: 021_012) and the Animal Research Ethics Committee at the University of KwaZulu Natal (reference number: AREC/00005911/2023). Additionally, authorization under Section 20 dispensation to perform animal research at UCT was granted by the South African Department of Agriculture, Land Reform and Rural Development [reference number: 12/11/1/7/1 (6151KL)]. All experimental procedures were conducted at the Institute of Infectious Diseases and Molecular Medicine at the UCT by researchers certified by the South African Veterinary Council.

### 2.2. Experimental Models

#### 2.2.1. Animals

All experiments in this study were conducted using female BALB/c mice (*n* = 6 per group; age, 6–10 weeks old; weight, 18–20 g). The mice were bred in-house under specific pathogen-free conditions at the UCT Research Animal Facility in South Africa. The mice were group-housed, with 6 mice per cage and food and water were provided *ad libitum*. A total of 24 mice were randomly sorted into four experimental groups, namely (1) singly infected with *Nb*, (2) singly infected with HSV-2, (3) co-infected with *Nb* and HSV-2, and (4) uninfected control groups.

All experimental procedures were conducted as previously described [17]. Firstly, to synchronize the estrous cycles, mice were subcutaneously treated with 2 mg Medroxyprogesterone Acetate (Depo Provera^®^) (Sigma-Aldrich, St. Louis, MO, USA) 7 days before the start of experimental procedures. Seven days later, mice were subcutaneously infected with 500 L3 *Nb* larvae. Seven days post-*Nb* infection, mice were infected with HSV-2 Strain G. All mice were sacrificed two days after HSV-2 challenge [17]. The FGT tissues (excluding ovaries) were isolated, preserved in RNAlater (Qiagen, Venlo, The Netherlands), kept at 4 °C overnight, and then stored at −80 °C until further analysis.

#### 2.2.2. *Nippostrongylus brasiliensis* (Nb) Maintenance and Infection

*Nb* was maintained in male Wistar rats as previously described [17]. Here, rats were subcutaneously infected with a dose of 5000 L3 *Nb* larvae. Following infection, stool samples were collected between days 6 to 8, which correspond with the peak production of helminth eggs. To prepare stool cultures, a mixture of stool and charcoal was placed on a moistened and raised filter paper to allow hatched L3 *Nb* larvae to migrate to the outer surface of the filter paper, where they were then collected by gently rinsing off with water. These L3 *Nb* larvae were counted under a dissecting microscope and suspended in an appropriate volume of distilled water for use in subsequent mice infections. In mice, infection with *Nb* was performed 7 days following Depo-Provera administration and 7 days before viral infection. This was performed once by subcutaneous infection using a 21G needle and a dose of 500 L3 *Nb* larvae in a total volume of 200 µL of sterile phosphate-buffered saline (PBS). Following infection with *Nb*, mice were monitored daily for changes in body weight and clinical pathology scores as indicators of infection and to ensure animal welfare.

#### 2.2.3. Virus

Human herpesvirus 2 strain G (HSV-2, ATCC, VR-734) was cultured in Vero cells at a multiplicity of infection (MOI) of 0.1, following previously described methods [17]. Two to three days later, both cells and supernatant were collected, and viral titers were measured by plaque assay. The viral aliquots were kept at −80 °C until further use.

For HSV-2 infection, mice were first anesthetized with Xylazine (10 mg/kg) + Ketamine (100 mg/kg) intraperitoneally using a 27G needle, and then infected with 5 × 10^5^ plaque-forming units (PFUs) of HSV-2 strain G in a total volume of 5 μL, intravaginally, using a Gilson P10 pipette and sterile 10 μL filter pipette tip. The pathogen was administered once. To collect vaginal lavages, the vaginal vaults were flushed ten times with 50 µL RNAlater; this step was performed three times. Following infection with HSV-2, mice were monitored daily for changes in body weight and clinical pathology scores as indicators of infection and to ensure animal welfare. Parameters included body weight, coat condition, breathing, mobility, evidence of barbering, and visible signs of inflammation at the sites of infection. The severity of HSV-2-associated illness was assessed by clinical characterization and scored from 0 to 5 as follows: 0—no pathology observed; 1—slight genital/perianal erythema; 2—genital/perianal swelling and erythema; 3—genital lesions and/or visible weight loss; 4—hind limb paralysis and/or purulent lesions; 5—premoribund [17].

### 2.3. Total RNA Extraction, Quantification, and Quality Control

The FGT tissues (excluding ovaries) of BALB/c mice were isolated, preserved in RNAlater (Qiagen, Venlo, The Netherlands), kept at 4 °C overnight, and then stored in a −80° C freezer until further analysis. FGT tissues were homogenized in 360 µL RPL buffer (Qiagen, Venlo, The Netherlands) on ice at 30 s intervals using a handheld homogenizer set on medium speed. Total RNA was extracted from the FGT tissues using the miRNeasy Tissue/Cells Advanced Micro Kit (Qiagen, Venlo, The Netherlands), which eliminates genomic DNA and yields RNA-enriched samples, according to the manufacturer’s instructions. Total RNA was eluted in a final volume of 15 µL RNAse-free water. To assess the concentration and quality of RNA samples, absorbances at 260 and 280 nm were measured with the NanoDrop 2000 spectrophotometer (Thermo Fisher Scientific, Waltham, MA, USA). Purified RNA samples were kept in a −80° C freezer.

### 2.4. Library Preparation and RNA Sequencing

RNA library preparation, quality assessment, and sequencing were performed by the South African Medical Research Council Genomics Platform (SAMRC Genomics Platform, Cape Town, South Africa). Poly (A) + RNA was isolated from total RNA using the Dynabeads mRNA Purification Kit (Thermo Fisher Scientific, Waltham, MA, USA). Libraries were constructed using the MGIEasy RNA Directional Library Prep Set (MGI Tech Co., Ltd., Shenzhen, China) according to the manufacturer’s instructions. The concentration and quality of both purified mRNA and constructed libraries were assessed by Qubit Fluorometer (Thermo Fisher Scientific, Waltham, MA, USA) and Agilent 2100 Bioanalyzer system (Agilent Technologies, Santa Clara, CA, USA), respectively. RNA integrity was confirmed using the RNA integrity number (RIN) via the Agilent 2100 Bioanalyzer system (Agilent, Santa Clara, CA, USA). All 24 RNA samples included in this study met the quality threshold, with RIN values greater than (>) 7. Sequencing was performed on the DNBSEQ-G400 system (MGI Tech Co., Ltd., Shenzhen, China) using a paired-end sequencing method (PE100) with the DNBSEQ-G400RS High-throughput Sequencing Set (FCL PE100) (MGI Tech Co., Ltd., Shenzhen, China).

### 2.5. Bioinformatics Analysis

Paired-end reads for the 24 samples were generated from the SAMRC Genomics platform and transferred to the Centre for High Performance Computing (CHPC) for subsequent data analysis. Bioinformatics analyses were performed by the DIstributed PLatform in OMICS (DIPLOMICS, Cape Town, South Africa). The RNA-sequencing data were processed using HTStream (v1.3.1) for quality control and preprocessing, which comprised removing adaptor sequences, contaminants, and low-quality reads to generate high-quality clean reads suitable for downstream analysis. A comprehensive MultiQC report was compiled using MultiQC (v1.21). No QC errors were encountered. RNA-sequencing reads were provided in fastq file format. Read alignment to the mouse reference genome was performed using STAR, and gene-level quantification was conducted. Normalization was carried out in R (version 4.3.3) using edgeR and limma packages.

### 2.6. Differentially Expressed Gene Analysis

The R packages, edgeR and limma, were used to analyse differential gene expression between two groups. Genes were considered significantly differentially expressed if they had an adjusted *p*-value less than (<) 0.05 and log fold change (LogFC) less than (<) −1 or greater than (>) 1, where logFC represents log2 fold change. Volcano plots and heatmaps were generated to visualize the expression profiles of differentially expressed genes (DEGs).

### 2.7. Identification of Immune-Related DEGs, Functional and Pathway Enrichment Analysis

To identify immune-related genes within the cohorts of DEGs, gene sets associated with immune functions were retrieved from the Mouse Genome Informatics (MGI) database. These gene sets were then intersected with the DEGs in each group-wise comparison. Functional enrichment analyses of the DEGs were performed using the online tool g: Profiler (URL: https://biit.cs.ut.ee/gprofiler/gost, accessed on 4 March 2025). The analyses encompassed Gene Ontology (GO) analysis, which included terms related to biological process (BPs), cellular component (CC), and molecular function (MF), Kyoto Encyclopaedia of Genes and Genomes (KEGG) pathway enrichment analysis, and Reactome pathway analysis. Statistical significance was assessed using the hypergeometric test or Fisher’s exact test, with multiple testing correction performed using the Benjamini–Hochberg false discovery rate (FDR) method. An FDR value < 0.05 was considered as significantly enriched. The online tool, SRplot (URL: https://www.bioinformatics.com.cn/en, accessed on 20 March 2025) was then used to visualize the DEG enrichment plots.

### 2.8. Protein–Protein Interaction Network Construction, Hub Gene and Functional Module Identification

For each group-wise comparison, the DEGs were uploaded to the Search Tool for the Retrieval of Interacting Genes/Proteins (STRING) database (version 12.0) (URL: https://string-db.org/, accessed on 2 April 2025) to predict protein–protein interaction (PPI) networks with a high confidence interaction score of 0.7. Each node in the network represents a target gene. Each edge connecting nodes indicates predicted interactions, with edge thickness reflecting interaction strength. Hub genes, which play key roles in the network, were identified using the CytoHubba plug-in in Cytoscape software (version 3.10.3). Hub genes were calculated using the maximal clique centrality (MCC) algorithm, which is considered a highly sensitive and specific algorithm for identifying hub genes [18]. The top 10 genes with the highest MCC scores were identified as hub genes. Cytoscape was then used to visualize hub gene networks, highlighting both the hub genes and their interactions. Functional modules within these networks were identified using the MCODE plug-in within Cytoscape.

## 3. Results

### 3.1. Animal Infection

This study examined gene expression profiles in murine FGT tissues across four experimental groups: (1) singly infected with *Nb*, (2) singly infected with HSV-2, (3) co-infected with both *Nb* and HSV-2, and (4) uninfected controls. Throughout the study, body weight and clinical pathology scores were monitored to track infection-associated changes. In *Nb*-infected mice, transient weight loss (<10%) was observed between days 3 and 4 post-infection, with complete recovery evident by day 9, as was anticipated. At experimental endpoint (day 2 post-HSV-2 infection), no overt clinical signs were observed, and there were no statistically significant differences in body weight or pathology scores among the four experimental groups, indicating no observable signs of distress or disease, as was anticipated (Supplementary Figure S1A,B).

### 3.2. Identification of DEGs

RNA-sequencing was employed to conduct an in-depth analysis of the FGT gene expression profiles of BALB/c mice with *Nb* and HSV-2 single or co-infection. Next, differential expression analysis (LogFC < −1 or >1, adjusted *p*-value < 0.05) of the group-wise comparisons of (a) uninfected versus *Nb*-infected FGT tissues, (b) uninfected versus HSV-2-infected FGT tissues, and (c) uninfected versus *Nb*/HSV-2 co-infected FGT tissues was performed.

**Figure 1:**
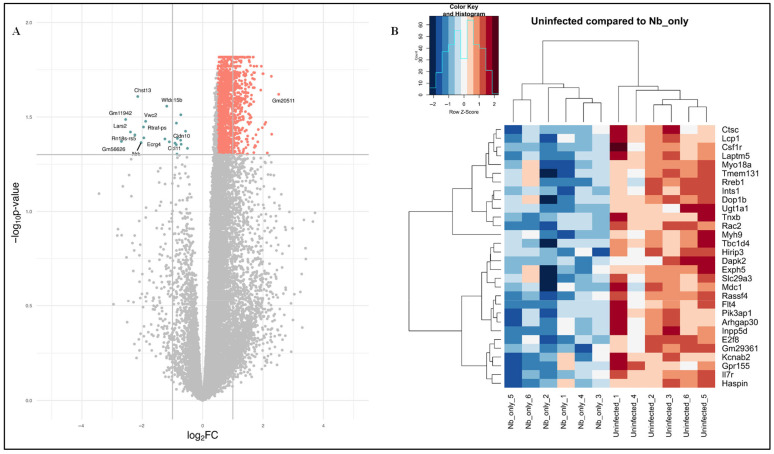
(**A**) Volcano plot of differentially expressed genes (DEGs) in uninfected versus *Nb*-infected FGT tissues. Orange dots represent significantly upregulated genes and blue dots represent significantly downregulated genes. Grey dots typically represent genes that do not meet the criteria for significant differential expression. (**B**) Heat map showing the top 30 DEGs in the comparison of uninfected versus *Nb*-infected FGT tissues. Z-score represents how much a gene’s expression level in a particular sample deviates from the average expression level of that gene across all samples, measured in terms of standard deviations. A positive Z-score indicates higher-than-average expression, while a negative Z-score indicates lower-than-average expression. The colours on the heat map correspond to the expression levels of DEGs across all the samples with the orange colour representing a higher expression than the mean and the blue colour representing a lower expression than the mean.

In the comparison of uninfected versus *Nb*-infected FGT tissues, a total of 368 DEGs were identified, of which 356 genes were upregulated and 12 genes were downregulated (Supplementary File S1). The top 30 DEGs are listed in Table 1. A volcano plot of the DEGs and heatmap illustrating the expression patterns of the top 30 DEGs between uninfected versus *Nb*-infected FGT tissues are shown in Figure 1A,B.

**Figure 2:**
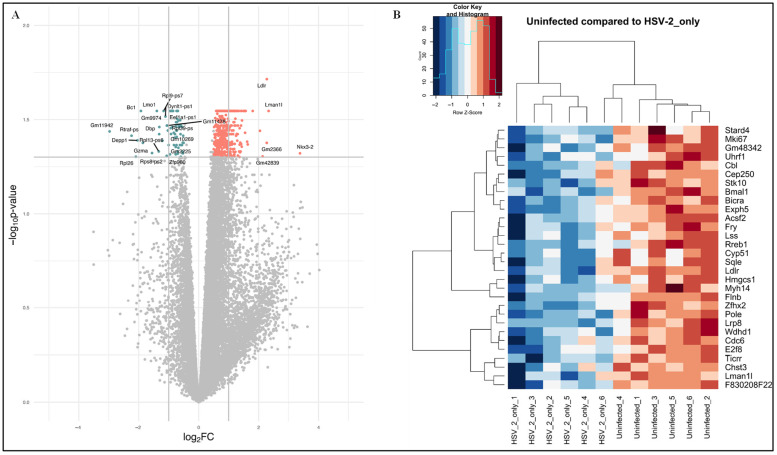
(**A**) Volcano plot of differentially expressed genes (DEGs) in uninfected versus HSV-2-infected FGT tissues. Orange dots represent significantly upregulated genes and blue dots represent significantly downregulated genes. Grey dots typically represent genes that do not meet the criteria for significant differential expression. (**B**) Heat map showing the top 30 DEGs in the comparison of uninfected versus HSV-2-infected FGT tissues. Z-score represents how much a gene’s expression level in a particular sample deviates from the average expression level of that gene across all samples, measured in terms of standard deviations. A positive Z-score indicates higher-than-average expression, while a negative Z-score indicates lower-than-average expression. The colours on the heat map correspond with the expression levels of DEGs across all the samples with the orange colour representing a higher expression than the mean and the blue colour representing a lower expression than the mean.

In the comparison of uninfected versus HSV-2-infected FGT tissues, a total of 140 DEGs were identified, of which 121 genes were upregulated and 19 genes were downregulated (Supplementary File S2). The top 30 DEGs are listed in Table 2. A volcano plot of DEGs and heatmap illustrating the expression patterns of the top 30 DEGs between uninfected versus HSV-2-infected FGT tissues are shown in Figure 2A,B.

No DEGs were identified in the group-wise comparison of uninfected versus *Nb*/HSV-2 co-infected FGT tissues. This group was subsequently excluded from downstream bioinformatics analyses.

### 3.3. Identification of Immune-Related DEGs

**Figure 3:**
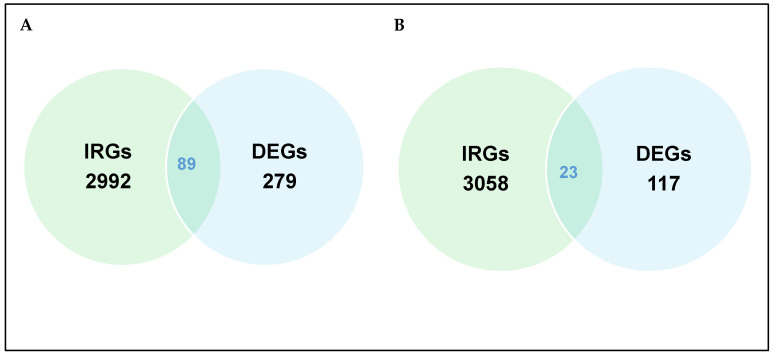
Venn diagram of total immune-related genes (IRGs) and differentially expressed genes (DEGs) in (**A**) uninfected versus *Nb*-infected FGT tissues and (**B**) uninfected versus HSV-2 FGT tissues.

To further identify immune-related genes within the cohorts of DEGs, gene sets associated with immune functions were retrieved from the Mouse Genome Informatics (MGI) database. These gene sets were then intersected with the DEGs in each group-wise comparison. In the uninfected versus *Nb*-infected FGT tissue comparison, 89 immune-related DEGs were obtained (Figure 3A, Table 3). In the uninfected versus HSV-2-infected FGT tissue comparison, 23 immune-related DEGs were obtained (Figure 3B, Table 3).

### 3.4. GO, KEGG and REACTOME Enrichment Analysis

**Figure 4:**
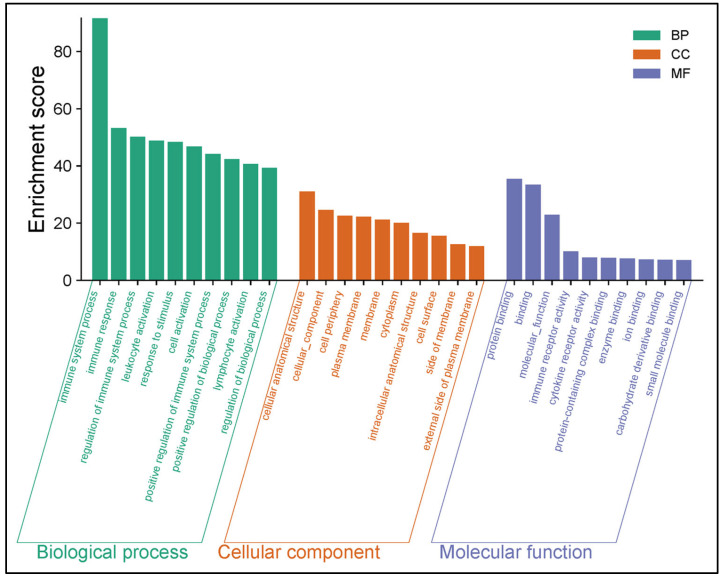
GO enrichment analysis of immune-related differentially expressed genes (DEGs) in the uninfected versus *Nb*-infected FGT tissues.

**Figure 5:**
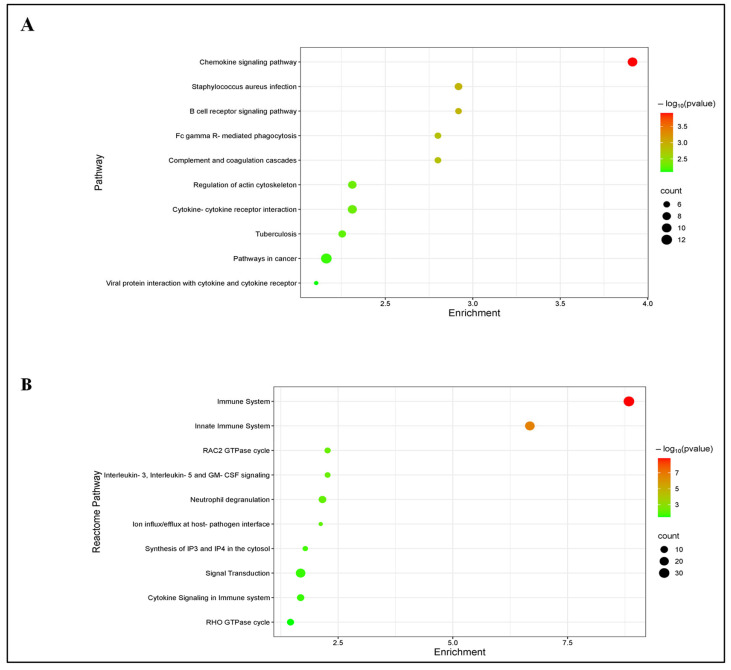
Immune-related differentially expressed genes (DEGs) in the uninfected versus *Nb*-infected FGT tissues: (**A**) KEGG enrichment analysis of immune-related DEGs and (**B**) REACTOME enrichment analysis of immune-related DEGs.

To elucidate the significant biological processes, functional classification and specific signaling pathways associated with the immune-related DEGs, GO, KEGG and REACTOME enrichment analyses were performed. In the uninfected versus *Nb*-infected FGT tissue comparison, the top five enriched GO BP terms included “immune system process”, “immune response”, “regulation of immune system process”, “leukocyte activation”, and “response to stimulus”. The top five GO CC terms were “cellular anatomical structure”, “cellular component”, “cell periphery”, “plasma membrane”, and “membrane”. The top five GO MF terms were “protein binding”, “binding”, “molecular function”, “immune receptor activity”, and “cytokine receptor activity” (Figure 4). In addition, KEGG enrichment analysis revealed that these DEGs were enriched in several immune-related pathways including “chemokine signaling pathway”, “B cell receptor signaling pathway”, “Fc gamma R-mediated phagocytosis”, “complement and coagulation cascades”, and “cytokine-cytokine receptor interaction” (Figure 5A). Similarly, REACTOME enrichment analysis revealed that the immune-related DEGs were enriched in “Immune System”, “Innate Immune System”, “RAC2 GTPase cycle”, “Interleukin-3, Interleukin-5 and GM-CSF signaling”, and “Neutrophil degranulation” (Figure 5B).

**Figure 6:**
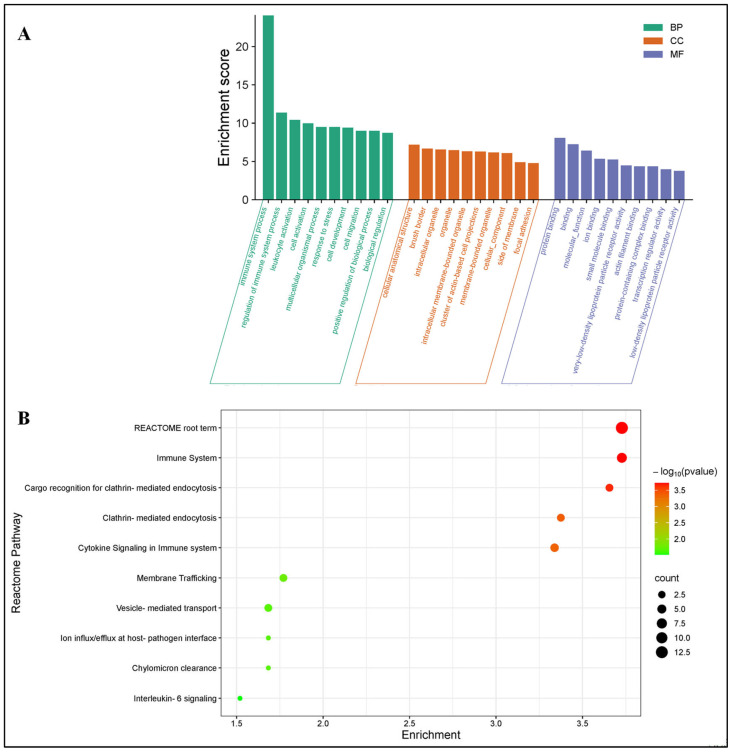
Immune-related differentially expressed genes (DEGs) in the uninfected versus HSV-2-infected FGT tissues: (**A**) GO enrichment analysis of immune-related DEGs and (**B**) REACTOME enrichment analysis of immune-related DEGs.

In the comparison of uninfected versus HSV-2-infected FGT tissues, the top five enriched GO BP terms for the immune-related DEGs included “immune system process”, “regulation of immune system process”, “leukocyte activation”, “cell activation”, and “multicellular organismal process”. The top five GO CC terms were “cellular anatomical structure”, “brush border”, “intracellular organelle”, “organelle”, and “intracellular membrane-bounded organelle”. The top five GO MF terms were “protein binding”, “binding”, “molecular function”, “ion binding”, and “small molecule binding” (Figure 6A). No enriched KEGG pathways were identified for these DEGs. The top REACTOME enriched pathways included “REACTOME root term”, “immune system”, “cargo recognition for clathrin-mediated endocytosis”, “clathrin-mediated endocytosis”, and “cytokine signaling in immune system” (Figure 6B).

Taken together, these findings indicate that immune-related DEGs were identified in comparisons between uninfected and infected FGT tissues (both *Nb*- and HSV-2-infected), and that these DEGs are involved in immunological and biological processes.

### 3.5. Protein–Protein Interaction (PPI) Network Analysis

To further explore PPI networks, hub genes, and key functional modules among all the DEGs identified in the group-wise comparisons, PPI network analysis was conducted using the STRING database. The resulting networks were visualized with Cytoscape software, and subsequently analysed for hub genes using the CytoHubba plug-in, and functional modules using the MCODE plug-in.

**Figure 7:**
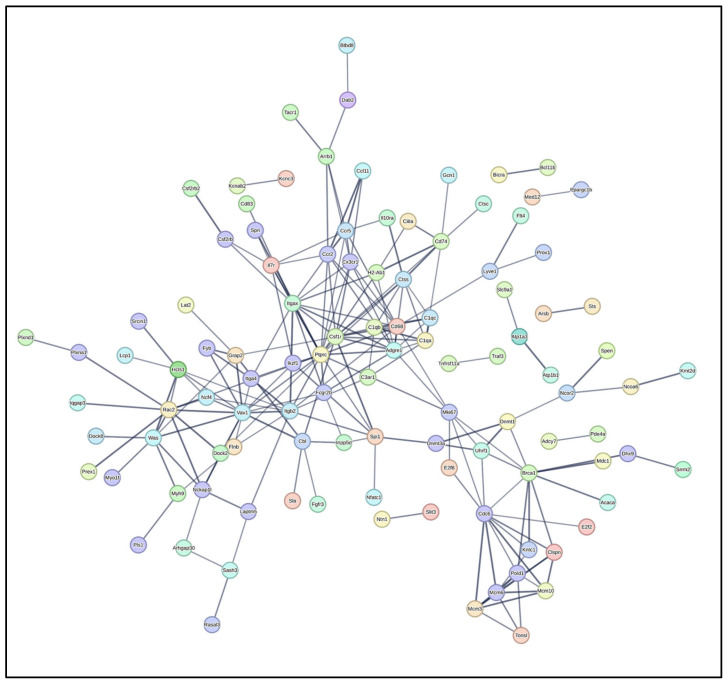
Differentially expressed genes (DEGs) analysed by protein–protein interaction (PPI) network. PPI network analysis was conducted on all DEGs in the uninfected versus *Nb*-infected FGT tissue comparison using STRING and the resulting network was visualized within Cytoscape. Colored nodes represent query proteins. Edges/lines represent functional associations. Solid lines indicate strong evidence or direct interactions. Edge thickness represents confidence score—thicker edges indicate higher confidence in interaction.

**Figure 8:**
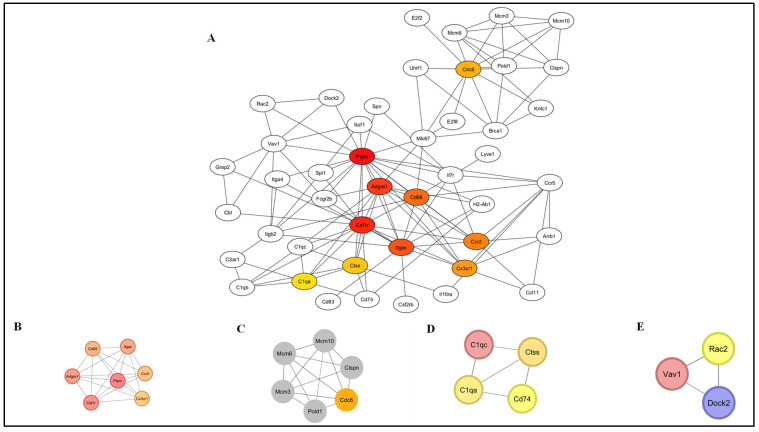
Differentially expressed genes (DEGs) analysed by protein–protein interaction (PPI) network. PPI network analysis was conducted on all DEGs in the uninfected versus *Nb*-infected FGT tissue comparison using STRING and the resulting network was visualized within Cytoscape. (**A**) Top 10 hub genes were identified using CytoHubba and (**B**–**E**) functional modules were identified using MCODE.

For the DEGs identified in the uninfected versus *Nb*-infected FGT tissue comparison, 291 nodes and 205 edges were obtained (PPI enrichment *p*-value < 1.0 × 10^−16^; confidence score > 0.7) (Figure 7). Hub genes were identified from the generated data in PPI network analysis, and the top 10 hub genes were obtained (*Ptprc*, *Csf1r*, *Adgre1*, *Itgax*, *Cd68*, *Ccr2*, *Cx3cr1*, *Cdc6*, *Ctss*, and *C1qa*) (Figure 8A). Moreover, four functional modules [Module 1 (Figure 8B), Module 2 (Figure 8C), Module 3 (Figure 8D), and Module 4 (Figure 8E)] were established from the PPI network using the MCODE plug-in. GO enrichment analysis was performed on the resulting functional modules. In terms of GO BP, it was shown that genes in Module 1 (*Ptprc*, *Adgre1*, *Csf1r*, *Cd68*, *Itgax*, *Ccr2*, and *Cx3cr1*) were significantly enriched in “regulation of macrophage migration”, “positive regulation of cell migration”, “tumor necrosis factor superfamily cytokine production”, “tumor necrosis factor production”, and “positive regulation of hematopoietic stem cell migration”. Module 2 genes (*Cdc6*, *Pold1*, *Mcm3*, *Mcm6*, *Mcm10*, and *Clspn*) were mainly enriched in “DNA-templated DNA replication”, “DNA replication initiation”, “DNA replication”, “DNA metabolic process”, and “DNA damage response”. Module 3 genes (*C1qc*, *Ctss*, *C1qa*, and *Cd74*) were enriched in “adaptive immune response”, “synapse pruning”, “antigen processing and presentation of exogenous peptide antigen via MHC class II”, “antigen processing and presentation of peptide antigen via MHC class II”, and “cell junction disassembly”. Module 4 genes (*Vav1*, *Rac2*, and *Dock2*) were enriched in “chemotaxis”, “taxis”, “T cell activation”, “neutrophil chemotaxis”, and “granulocyte chemotaxis” (Supplementary File S3).

**Figure 9:**
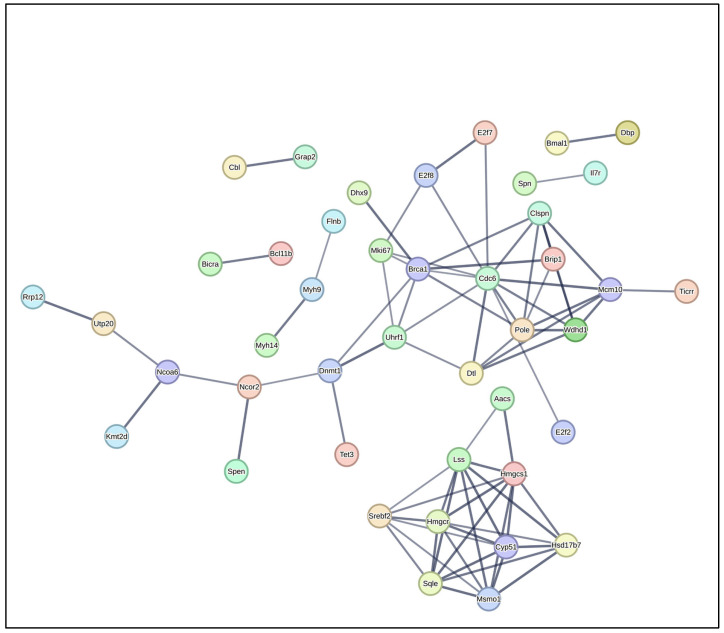
Differentially expressed genes (DEGs) analysed by protein–protein interaction (PPI) network. PPI network analysis was conducted on all DEGs in the uninfected versus HSV-2-infected FGT tissue comparison using STRING and the resulting network was visualized within Cytoscape. Colored nodes represent query proteins. Edges/lines represent functional associations. Solid lines indicate strong evidence or direct interactions. Edge thickness represents confidence score—thicker edges indicate higher confidence in interaction.

**Figure 10:**
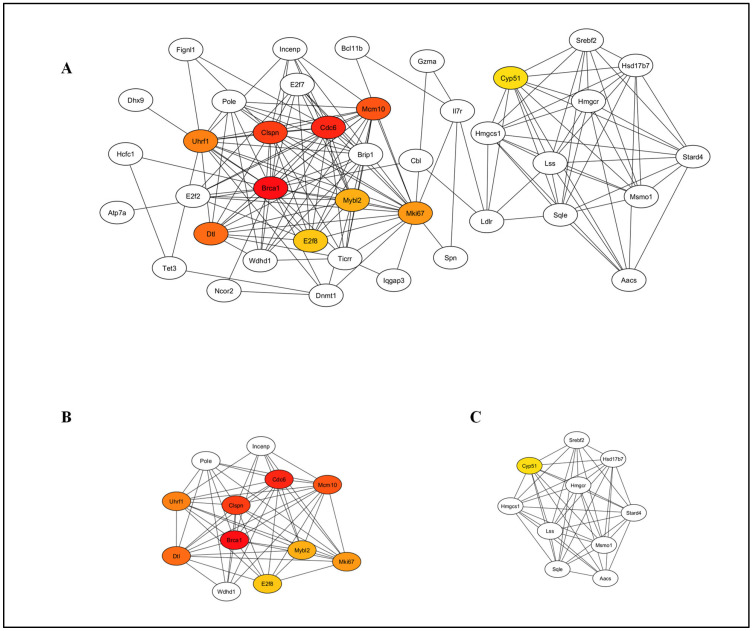
Differentially expressed genes (DEGs) analysed by protein–protein interaction (PPI) network. PPI network analysis was conducted on all DEGs in the uninfected versus HSV-2-infected FGT tissue comparison using STRING and the resulting network was visualized within Cytoscape. (**A**) Top 10 hub genes were identified using CytoHubba and (**B**,**C**) functional modules were identified using MCODE.

For the DEGs identified in the uninfected versus HSV-2 infected FGT tissue comparison, 103 nodes and 78 edges were obtained (PPI enrichment *p*-value < 1.0 × 10^−16^; confidence score > 0.7) (Figure 9). Hub genes were identified from the generated data in PPI network analysis, and the top 10 hub genes were identified (*Brca1*, *Cdc6*, *Clspn*, *Mcm10*, *Dtl*, *Uhrf1*, *Mki67*, *Mybl2*, *E2f8*, and *Cyp51*) (Figure 10A). Moreover, two functional modules [Module 1 (Figure 10B) and Module 2 (Figure 10C)] were established from the PPI network with the MCODE plug-in. In addition, GO enrichment analysis was performed on the resulting functional modules. In terms of GO BP, it was shown that genes in Module 1 (*Brca1*, *Cdc6*, *Clspn*, *Mcm10*, *Dtl*, *Uhrf1*, *Mki67*, *Mybl2*, *E2f8*, *Incenp*, *Pole*, and *Wdhd1*) were mainly enriched in “mitotic cell cycle”, “mitotic cell cycle process”, “cell cycle”, “cell cycle process” and “DNA replication”. Module 2 genes (*Cyp51*, *Lss*, *Srebf2*, *Hsd17b7*, *Stard4*, *Aacs*, *Sqle*, *Hmgcs1*, *Hmgcr*, and *Msmo1*) were mainly enriched in “cholesterol metabolic process”, “sterol biosynthetic process”, “sterol metabolic process”, “secondary alcohol metabolic process”, and “steroid biosynthetic process” (Supplementary File S4).

In summary, bioinformatic analyses predicted that the hub genes and key functional modules among all the DEGs identified in group-wise comparisons are associated with immune responses and host defense mechanisms against pathogens.

## 4. Discussion

In this preliminary study, we employed NGS and bioinformatics tools to identify DEGs and their associated immunological and biological functions in murine FGT tissues in the setting of *Nb* and HSV-2 single and co-infection. Our findings demonstrate the value of this approach in elucidating underlying molecular pathways potentially involved in host immune responses, while also revealing common and distinct features related to both pathogens.

### 4.1. Animal Infection

In this study, gene expression profiles in murine FGT tissues across four experimental groups were examined: (1) singly infected with *Nb*, (2) singly infected with HSV-2, (3) co-infected with both *Nb* and HSV-2, and (4) uninfected controls. For the duration of the study, body weight and clinical pathology scores were monitored to track infection-associated changes and ensure animal welfare. *Nb* infection dynamics are well-characterized and have been validated in previous studies; clinical symptoms are generally mild and brief, with the infection resolving within 10 days [17,19]. In *Nb*-infected mice, the transient weight loss observed around days 3–4 corresponds with the helminth’s pulmonary migration phase and resolved as *Nb* migrated to the intestine, consistent with the parasite’s natural lifecycle [19]. Similarly, following intravaginal infection of murine models with HSV-2, clinical symptoms are typically observed from day 3 onwards [17,20]. In our study, the absence of observable pathology at day 2 post-HSV-2 infection is consistent with previous findings, which indicate that early-stage HSV-2 infection in murine models causes minimal clinical symptoms [17]. Moreover, this early time point allowed us to capture transcriptional and immunological changes prior to the onset of overt pathology.

### 4.2. DEGs Identified in the Comparison of Uninfected Versus Nb-Infected FGT Tissues

Our comparative analysis of uninfected and *Nb*-infected FGT tissues identified 368 DEGs, comprising 356 upregulated and 12 downregulated genes. Among these, 89 DEGs were functionally annotated as immune-related based on MGI database comparisons, including key complement system components (e.g., *C3ar1*, *C1qa*, *C1qb*, *C1qc*). The complement system plays a vital role in innate immunity through opsonization, chemotaxis, and membrane attack complex formation, while also bridging innate and adaptive immune responses [21]. Consistent with these findings, GO enrichment analysis demonstrated significant representation of immune system processes among the DEGs, with 81 genes (e.g., *Rac2*, *Pik3ap1*, *Myo18a*, *Il7r*, *Laptm5*, *Csf1r*, *Ctsc*, *Dapk2*, *Inpp5d*, and *Myh9*) associated with immune-related processes. This finding was further supported by KEGG and Reactome pathway analyses, which revealed enrichment in critical immune pathways, including chemokine signaling and B-cell receptor signaling.

PPI network analysis identified ten hub genes central to immune function (*Ptprc*, *Csf1r*, *Adgre1*, *Itgax*, *Cd68*, *Ccr2*, *Cx3cr1*, *Cdc6*, *Ctss*, *C1qa*). Functional module analysis further confirmed immune-related roles for three key modules: (1) Module 1 (*Ptprc*, *Adgre1*, *Csf1r*, *Cd68*, *Itgax*, *Ccr2*, *Cx3cr1*), (2) Module 3 (*C1qc*, *Ctss*, *C1qa*, *Cd74*), and (3) Module 4 (*Vav1*, *Rac2*, *Dock2*).

Interestingly, we observed a predominant downregulation of immune-related DEGs in *Nb*-infected FGT tissues. This potentially reflects a complex interplay between various mechanisms, such as (1) FGT-specific factors related to hormonal and microbiota influences [22,23], and (2) immune modulation by the hookworm, downregulating pro-inflammatory/Th1 pathways in favour of a Th2/anti-inflammatory environment [11]. While beneficial for hookworm survival, this immune modulation may compromise critical FGT-specific mucosal defenses against secondary infections by impairing Th1 responses and epithelial protection mechanisms [4].

In addition, we identified Th2-associated chemokines (e.g., *Ccl11*) and receptors (e.g., *Ccr2* and *Cx3cr1*), which orchestrate leukocyte recruitment in normal and inflammatory conditions [24]. *Ccl11*, together with *Ccl24* and *Ccl26*, serves as a potent eosinophilic chemoattractant via *Ccr3* signaling [25]. These chemokines are induced by Th2 cytokines (IL-4 and IL-13) and work synergistically with IL-5 to drive eosinophilia [24], a hallmark of helminth infections [26]. Our observation of elevated *Ccl11* in *Nb*-infected FGT tissues aligns with previous reports of eosinophil accumulation in the murine FGT post-*Nb* infection [17], and mirrors established gastrointestinal responses to helminth infections [27,28].

The chemokine receptor *Ccr2* interacts with multiple CC chemokines (*Ccl2*, *Ccl7*, *Ccl8*, *Ccl12*, *Ccl13*, and *Ccl16*), with the *Ccl2-Ccr2* axis being important for monocyte recruitment during inflammatory responses, and recruitment of lymphocytes, NK cells, and other leukocytes [24]. In helminth infections, this axis is crucial for Th2 responses, as was demonstrated in *Schistosoma mansoni*-infected *Ccr2*-deficient mice, which exhibited defective monocytes and macrophages, and reduced IL-4 production [29]. Elevated *Ccl2* levels have been linked to strong Th2 immune responses, as observed in STH infections including *Trichuris muris* [30], *Ascaris suum* [31], and *Nb* [32]. Notably, *Ccl2*-deficient mice failed to expel *Trichuris muris* due to weakened Th2 (decreased IL-4) and heightened Th1 (elevated IFN-γ/IL-12) immune responses [33].

We observed a downregulation of *Cx3cr1* in *Nb*-infected FGT tissues. *Cx3cr1*, a receptor for *Cx3cl1* (fractalkine), mediates monocyte adhesion and tissue migration, where they can differentiate into dendritic cells and macrophages [25]. In *Nb* infection, *Cx3cr1*-deficient mice exhibited reduced pathology and improved parasite control, suggesting that *Cx3cr1* impairs effective monocyte–helminth interactions [34]. *Cx3cr1* was also expressed on a subset of CD4^+^ T cells that accumulated in helminth-infected tissues, indicating its role in Th2 immunity and tissue repair [35]. These findings underscore dual roles for *Cx3cr1* in monocyte and T cell responses during helminth infections.

Overall, our analyses demonstrate that *Nb* induces a robust immune response, with notable downregulation of immune-related genes in the FGT, a site distal to its primary site of infection. This is a significant finding given the hookworm’s lifecycle, which involves penetration of the skin, a transient migratory phase through the lungs, and establishment in the intestine [19,36]. The gene expression signature observed in our study is consistent with a coordinated Th2 immune response, involving leukocyte activation, chemokine-driven recruitment, macrophage and T-cell regulation. These findings align with reported Th2 signatures in the FGTs of STH-infected mice [17] and women [14,16].

### 4.3. DEGs Identified in Comparison of Uninfected Versus HSV-2-Infected FGT Tissues

Our comparative analysis of uninfected and HSV-2-infected FGT tissues identified 140 DEGs, comprising 121 upregulated and 19 downregulated genes. Intersection with immune-related gene sets from the MGI database revealed 23 immune-associated DEGs. GO analysis demonstrated significant enrichment of these DEGs in immune system processes. For instance, 22 genes (e.g., *Ldlr*, *Camk1d*, *Lrp8*, *Epg5*, *Myo18a*, *Flnb*, *Lmo1*, *Cbl*, *Stk10*, *Chst3*) were assigned to this functional category. Reactome pathway analysis further confirmed the involvement of these DEGs in key immunological pathways, including immune system function and cytokine-mediated signaling.

Notably, we observed that HSV-2-infected FGT tissues exhibited predominant downregulation of immune-related DEGs at 2 days post-infection. This early transcriptional suppression is consistent with known HSV-2 immune evasion mechanisms, including host gene shutoff, mRNA degradation, and interferon signaling inhibition [37]. This observed suppression likely reflects viral disruption of both antigen presentation and innate immune pathway activation [38,39,40]. For example, molecular studies demonstrated that HSV-2 viral proteins actively suppress host immune responses: HSV-2 US1 inhibited IFN-β production by preventing IRF-3 from binding to the IRF-3 response element within the IFN-β promoter region [38], ICP22 significantly inhibited IFN-β induction and functioned as an E3 ubiquitin-protein ligase to block IFN-stimulated gene production [40], and ICP27 similarly suppressed IFN-β generation [39]. Furthermore, the observed transcriptional profile may reflect the temporal dynamics of HSV-2 infection, as murine models have demonstrated that robust immune cell infiltration, particularly dendritic cells, macrophages, and T cells, and inflammatory gene expression typically peaked 3–5 days post-infection [41,42]. Therefore, the delay between initial viral replication and subsequent immune activation may account for the prevalence of downregulated genes in our early time point analysis.

PPI network analysis of hub genes and functional modules revealed no direct associations with immune responses or inflammation. Instead, the predominant functional annotations were related to mitotic cell cycle regulation, DNA replication initiation, and sterol biosynthesis.

The top hub gene, *Brca1*, is primarily recognized for its role in DNA repair and genomic stability [43]. While viral infections, including HCMV and HSV-1, have been shown to modulate *Brca1* expression [44], no such relationship has been described for HSV-2.

Notably, we identified multiple genes involved in (1) cell cycle (*Cdc6*, *Clspn*, *Mcm10*, *Dtl*, *Uhrf1*, *Mki67*, *Mybl2*, *E2f8*, *Incenp*, *Pole*, and *Wdhd1*), (2) DNA replication (*Cdc6*, *Mcm10*, *Pole* and *Wdhd1*), (3) cell cycle regulation (*Dtl*, *Mybl2*, *E2f8*), (4) DNA damage response (*Clspn*), (5) cytokinesis (*Incenp*), (6) chromatin organisation (*Uhrf1*), and (7) chromosome partition (*Mki67*) [45]. The downregulation of these genes in HSV-2-infected FGT tissues suggests viral manipulation of host cell cycle machinery. HSV-2 is known to hijack cellular replication pathways, particularly by promoting G1/S transition to access DNA synthesis machinery [46,47]. Recent evidence indicates that HSV-2 UL24 protein modulates cell cycle progression, while its N-terminal domain (UL24-N) specifically suppresses IFN-β production by inhibiting IRF-3 phosphorylation [48].

We identified several genes that regulate cholesterol biosynthesis and sterol metabolism (*Cyp51*, *Lss*, *Srebf2*, *Hsd17b7*, *Stard4*, *Aacs*, *Sqle*, *Hmgcs1*, *Hmgcr*, *Msmo1*). Cellular cholesterol plays a critical role in HSV infection, facilitating viral entry, nuclear transport of viral capsids, membrane fusion, and cell-to-cell spread [49,50]. Cholesterol depletion impaired viral protein expression, genome encapsidation, and virion release [50].

The sterol regulatory element-binding proteins (SREBPs), particularly SREBP2 encoded by *SREBF2*, are key regulators of lipid homeostasis [51]. Beyond lipid metabolism, SREBP2 influenced immune responses by activating the NLRP3 inflammasome in endothelial cells [52], regulated chemokine production through direct transcriptional activation of BHLHE40 and KLF6 [53], and antagonized type I IFN responses [54]. Notably, type I IFN signaling suppressed cholesterol synthesis and promoted fatty acid import, creating an antiviral state [54]. Taken together, this metabolic/immunological crosstalk suggests that HSV-2-mediated modulation of sterol metabolism contributes to both viral strategy for replication and host defense.

### 4.4. Limitations of the Current Study and Future Directions

A key objective of this study was to characterize FGT transcriptional changes in response to *Nb*/HSV-2 co-infection. Notably, our comparative analysis revealed no significant DEGs between uninfected and co-infected FGT tissues, which contrasts with the distinct transcriptional changes observed in single *Nb* and HSV-2 infections. It is plausible that this finding is biologically relevant, potentially reflecting immune antagonism, whereby *Nb*-induced Th2 and immunomodulatory responses downregulate early HSV-2-mediated Th1/pro-inflammatory responses [11], resulting in transcriptional equilibrium. We emphasize that while this finding requires validation in future studies, it underscores the need to study STH/HSV-2 co-infections further to elucidate complex immunological interactions. However, we also acknowledge that subtle transcriptional changes in the co-infected FGT tissues may not have been detected due to the small sample size (*n* = 6 per group). Given that this was a proof-of-concept study, the sample size was selected based on previously established methods [17], while adhering to the ethical principles governing animal research [55]. We assert that future studies should (1) use larger cohort sizes to improve statistical power, (2) compare acute versus chronic infection stages, (3) examine diverse STH species to assess species-specific effects on HSV-2 co-infection, and (4) validate the functional roles of identified DEGs through mechanistic studies. Despite these limitations, our findings provide novel insights into FGT transcription changes during single *Nb* and HSV-2 infections, enabling an enhanced understanding of host–pathogen interactions. Our study highlights NGS as a powerful tool for elucidating the molecular mechanisms of *Nb* and HSV-2 infections in FGT tissues. The transcriptional profiles generated through RNA-sequencing provide a foundation for future investigations to develop novel diagnostic biomarkers distinguishing single versus co-infections and identify potential therapeutic targets for these clinically relevant infections.

## 5. Conclusions

Our study highlights the value of NGS technology in exploring complex immunological responses to *Nb*, HSV-2 and their co-infection. The data generated through NGS enabled detailed analyses of gene expression profiles and immune-related pathways in the murine FGT, revealing previously unappreciated immune-related genes and unique transcriptional profiles. The findings of our study lay a foundation for future investigations into the molecular mechanisms underlying STH and HSV-2 infections, with the goal of enhancing diagnostic tools and therapeutic interventions, which is especially relevant in co-endemic regions where both pathogens are highly prevalent.

## Figures and Tables

**Table 1:** Top 30 significant DEGs in the comparison of uninfected versus *Nb*-infected female genital tract tissues.

Gene ID	Gene Name	logFC	*p* Value	Adjusted *p* Value
ENSMUSG00000033016.17	*Nfatc1*	1.01	2.30 × 10^−6^	0.02
ENSMUSG00000033220.8	*Rac2*	1.30	2.60 × 10^−6^	0.02
ENSMUSG00000025017.11	*Pik3ap1*	1.55	6.32 × 10^−6^	0.02
ENSMUSG00000029098.18	*Acox3*	1.02	9.09 × 10^−6^	0.02
ENSMUSG00000038644.15	*Pold1*	1.02	1.63 × 10^−5^	0.02
ENSMUSG00000039637.16	*Coro7*	1.05	1.73 × 10^−5^	0.02
ENSMUSG00000033083.17	*Tbc1d4*	1.11	1.85 × 10^−5^	0.02
ENSMUSG00000089960.2	*Ugt1a1*	1.27	2.14 × 10^−5^	0.02
ENSMUSG00000029547.11	*Ints1*	1.16	2.14 × 10^−5^	0.02
ENSMUSG00000041638.19	*Gcn1*	1.04	2.57 × 10^−5^	0.02
ENSMUSG00000000631.21	*Myo18a*	1.14	3.02 × 10^−5^	0.02
ENSMUSG00000003882.6	*Il7r*	1.54	3.37 × 10^−5^	0.02
ENSMUSG00000032536.13	*Trak1*	1.08	3.37 × 10^−5^	0.02
ENSMUSG00000028581.18	*Laptm5*	1.68	4.39 × 10^−5^	0.02
ENSMUSG00000004099.17	*Dnmt1*	1.06	4.92 × 10^−5^	0.02
ENSMUSG00000034584.4	*Exph5*	1.56	5.28 × 10^−5^	0.02
ENSMUSG00000031004.9	*Mki67*	1.05	5.45 × 10^−5^	0.02
ENSMUSG00000024621.17	*Csf1r*	1.68	5.55 × 10^−5^	0.02
ENSMUSG00000026116.12	*Tmem131*	1.10	5.85 × 10^−5^	0.02
ENSMUSG00000030560.18	*Ctsc*	1.32	6.20 × 10^−5^	0.02
ENSMUSG00000022946.11	*Dop1b*	1.12	7.49 × 10^−5^	0.02
ENSMUSG00000032380.10	*Dapk2*	1.37	7.63 × 10^−5^	0.02
ENSMUSG00000020357.4	*Flt4*	1.48	7.64 × 10^−5^	0.02
ENSMUSG00000061607.16	*Mdc1*	1.09	8.16 × 10^−5^	0.02
ENSMUSG00000028931.13	*Kcnab2*	1.47	8.23 × 10^−5^	0.02
ENSMUSG00000120290.1	*Gm56959*	1.08	8.40 × 10^−5^	0.02
ENSMUSG00000026288.15	*Inpp5d*	1.67	8.97 × 10^−5^	0.02
ENSMUSG00000022443.18	*Myh9*	1.29	9.65 × 10^−5^	0.02
ENSMUSG00000020100.16	*Slc29a3*	1.16	9.69 × 10^−5^	0.02
ENSMUSG00000048865.17	*Arhgap30*	1.50	9.84 × 10^−5^	0.02

**Table 2:** Top 30 significant DEGs in the comparison of uninfected versus HSV-2-infected female genital tract tissues.

Gene ID	Gene Name	logFC	*p* Value	Adjusted *p* Value
ENSMUSG00000032193.10	*Ldlr*	2.27	9.96 × 10^−7^	0.02
ENSMUSG00000031004.9	*Mki67*	1.24	5.82 × 10^−6^	0.03
ENSMUSG00000039145.17	*Camk1d*	1.14	8.31 × 10^−6^	0.03
ENSMUSG00000004356.9	*Utp20*	1.04	9.39 × 10^−6^	0.03
ENSMUSG00000046179.18	*E2f8*	1.53	1.28 × 10^−5^	0.03
ENSMUSG00000024251.11	*Thada*	1.02	1.44 × 10^−5^	0.03
ENSMUSG00000028613.16	*Lrp8*	1.28	2.26 × 10^−5^	0.03
ENSMUSG00000055116.9	*Bmal1*	1.27	2.49 × 10^−5^	0.03
ENSMUSG00000024660.10	*Incenp*	1.01	2.54 × 10^−5^	0.03
ENSMUSG00000114934.2	*Gm48342*	1.52	3.35 × 10^−5^	0.03
ENSMUSG00000024378.10	*Stard4*	1.25	3.38 × 10^−5^	0.03
ENSMUSG00000093930.3	*Hmgcs1*	1.18	4.20 × 10^−5^	0.03
ENSMUSG00000004099.17	*Dnmt1*	1.04	5.12 × 10^−5^	0.03
ENSMUSG00000073529.10	*F830208F22Rik*	1.80	5.91 × 10^−5^	0.03
ENSMUSG00000022351.15	*Sqle*	1.42	6.44 × 10^−5^	0.03
ENSMUSG00000039835.17	*Nhsl1*	1.07	6.59 × 10^−5^	0.03
ENSMUSG00000039840.9	*Epg5*	1.11	6.98 × 10^−5^	0.03
ENSMUSG00000000631.21	*Myo18a*	1.06	7.31 × 10^−5^	0.03
ENSMUSG00000030739.20	*Myh14*	1.23	7.35 × 10^−5^	0.03
ENSMUSG00000021670.15	*Hmgcr*	1.05	7.51 × 10^−5^	0.03
ENSMUSG00000035049.5	*Rrp12*	1.13	8.00 × 10^−5^	0.03
ENSMUSG00000039087.18	*Rreb1*	1.17	8.25 × 10^−5^	0.03
ENSMUSG00000022463.9	*Srebf2*	1.02	8.47 × 10^−5^	0.03
ENSMUSG00000013629.17	*Cad*	1.02	8.78 × 10^−5^	0.03
ENSMUSG00000001228.15	*Uhrf1*	1.18	9.33 × 10^−5^	0.03
ENSMUSG00000046591.11	*Ticrr*	1.43	9.35 × 10^−5^	0.03
ENSMUSG00000025278.10	*Flnb*	1.17	9.79 × 10^−5^	0.03
ENSMUSG00000040721.10	*Zfhx2*	1.22	9.81 × 10^−5^	0.03
ENSMUSG00000115783.3	*Bc1*	−1.93	9.84 × 10^−5^	0.03
ENSMUSG00000034584.4	*Exph5*	1.42	1.01 × 10^−4^	0.03

**Table 3:** Immune-related DEGs in the uninfected versus (A) *Nb*-infected and (B) HSV-2-infected FGT tissues.

(A) Immune-Related DEGs: Uninfected Versus *Nb*-Infected FGT Tissues	(B) Immune-Related DEGs: Uninfected Versus HSV-2-Infected FGT Tissues
*Nfatc1*, *Rac2*, *Pik3ap1*, *Myo18a*, *Il7r*, *Laptm5*, *Csf1r*, *Ctsc*, *Dapk2*, *Inpp5d*, *Myh9*, *Lcp1*, *Spn*, *Dock2*, *Itgb2*, *Cd74*, *Itgax*, *Nckap1l*, *Wdfy4*, *Csf2rb2*, *Ccr5*, *Ciita*, *Apbb1ip*, *Stk10*, *Cx3cr1*, *Chst3*, *Hcls1*, *Crtc3*, *Ptpro*, *Flnb*, *Ppargc1b*, *H2-Ab1*, *Cd180*, *Cbl*, *Adcy7*, *Vav1*, *Traf3*, *Pigr*, *Slc11a1*, *Dock8*, *Ccdc88b*, *Mpeg1*, *Zmiz1*, *C1qc*, *Prex1*, *Dtx1*, *Atp7a*, *Myo1f*, *Trim56*, *Elf4*, *Fgfr3*, *Trim62*, *Pld4*, *Bcl11b*, *Itga4*, *C1qb*, *Ctss*, *Wfdc15b*, *Tnfrsf11a*, *Cd68*, *Was*, *Ptprc*, *Adgre1*, *Ccr2*, *Dhx9*, *Lat2*, *C3ar1*, *Fcgr2b*, *Rab43*, *Naip2*, *Cd83*, *Tifab*, *Lyve1*, *Msmp*, *Plxna1*, *Spi1*, *Dtx4*, *Ikzf1*, *Itpkb*, *Rasal3*, *Csf2rb*, *Cd84*, *Sash3*, *Ccl11*, *C1qa*, *Myo1g*, *Epas1*, *Themis2*, *Tacr1*	*Ldlr*, *Camk1d*, *Lrp8*, *Epg5*, *Myo18a*, *Flnb*, *Lmo1*, *Cbl*, *Stk10*, *Chst3*, *Bcl11b*, *Il7r*, *Spn*, *Atp7a*, *Foxn1*, *Zmiz1*, *Zbtb34*, *Dhx9*, *Myh9*, *Plec*, *Csf2rb2*, *Nkx3-2*, *Tnfrsf11a*

## Data Availability

The data underlying the findings have been included in the article and Supplementary Material. Further inquiries may be directed to the corresponding author.

## Supplementary Materials

The following supporting information can be downloaded at https://www.mdpi.com/article/10.3390/pathogens14080795/s1, Supplementary Figure S1A: Daily weights were assessed between the groups post infection; Supplementary Figure S1B: Pathology scoring was assessed between the groups post infection; Supplementary File S1: List of Significant Differentially Expressed Genes Identified in the uninfected versus Nb-infected comparison; Supplementary File S2: List of Significant Differentially Expressed Genes Identified in the uninfected versus HSV-2-infected comparison; Supplementary File S3: GO Analysis of Functional Modules: Uninfected FGT tissues versus Nb-infected FGT tissues; Supplementary File S4: GO Analysis of Functional Modules: Uninfected FGT tissues versus HSV-2-infected FGT tissues.
